# The Value and Challenges of Implementing Patient Centered Measures in a Psychiatric Hospital Setting

**DOI:** 10.1192/j.eurpsy.2022.69

**Published:** 2022-09-01

**Authors:** E. Scanferla, P. Gorwood

**Affiliations:** 1 University of Paris, Cmme, Ghu Paris Paris Psychiatry And Neurosciences, Paris, France; 2 University of Paris, Cmme, Hopital Sainte-anne Ghu Paris Psychiatrie Et Neurosciences, Paris, France

**Keywords:** Patient-reported outcome measures (PROMs), Patient-reported experience measures (PREMs), Value-Based Healthcare, Quality of Hospital Care

## Abstract

Background: Measuring and interpreting outcome is challenging in mental health services than in some other areas of health care. Objectives: The aims of this study were to (1) explore results of Patient-Reported Outcome Measures (PROMs) and Patient-Reported Experience Measures (PREMs) in psychiatric hospital settings, (2) describe the relation between generic PROMs (measure of well-being) and specific PROMs (disorder-specific symptom assessments), (3) describe the congruence between patients and clinicians evaluation of the care experience (based on Patients reported experience measures and Clinician reported experience measures). Methods: A total of 269 consecutive patients participated in this study. Results: (1) Subjective experience of well-being (outcome) improved after hospitalisation (+15%, avg). High satisfaction with subjective experience of care (85%, avg). (2) Significant correlation between patients’ assessment of subjective well-being (generic PROMs) and clinical improvement (specific PROMs) (p <0.007). (3) Significant correlation between patient and clinician experience of care (p= 0.002). Conclusions: One of the first French studies on the use of standardised PROMs and PREMs in psychiatric hospital settings. Results suggest that subjective well-being measures complement the assessment of the patient’s clinical symptoms and social functioning. The effectiveness of care depends on the consideration of these three dimensions. The use of core patient-reported measures, as part of systematic measurement and performance monitoring in mental health care, provides valuable input to the clinicians’ practice.

**Disclosure:**

No significant relationships.

